# P03-04 Perceived and objective indicators of neighbourhood safety and physical activity in early adolescence: a national cohort study

**DOI:** 10.1093/eurpub/ckac095.040

**Published:** 2022-08-29

**Authors:** Charlotte Constable Fernandez, Praveetha Patalay, Jane Maddock, Laura Vaughan

**Affiliations:** UCL Institute of Cardiovascular Science, London, United Kingdom

**Keywords:** physical activity, adolescence, neighbourhoods, crime, safety

## Abstract

**Background:**

The health benefits of regular physical activity in adolescence are well-documented. Currently, young people in the UK are not achieving recommended levels of physical activity. The neighbourhood environment is a key setting for physical activity in adolescence. Due to lack of financial independence and mobility restrictions, adolescents spend a significant amount of time in their neighbourhood. Feeling unsafe in their neighbourhood may be a potential barrier to physical activity.

This study aims to examine associations between objective and subjective measures of neighbourhood safety and physical activity.

**Methods:**

Participants (n = 11,726) came from the Millennium Cohort Study; a nationally representative UK longitudinal birth cohort. At age 11 perceived neighbourhood safety was assessed via questionnaire and the Index of Multiple Deprivation (IMD) crime domain was linked to participant postcode data. At age 14, participants self-reported physical activity and a subsample (n = 4,813) also wore GENEActiv wrist-worn activity monitors for one weekday and one weekend day. Associations between perceived safety, IMD crime and self-reported physical activity were quantified using linear regression models. Zero Inflated Poisson (ZIP) models were used to examine associations with accelerometer-measured physical activity. We adjusted for parental education, family income, ethnicity and season of accelerometer wear. An interaction term for sex was tested to assess whether associations between perceived safety and physical activity differed by sex. Models were also stratified by sex.

**Results:**

Feeling not safe compared to very safe was associated with 0.28 (95% CI -0.50, -0.06) fewer days of self-reported physical activity. However, no association was seen between perceived safety and accelerometer physical activity. Those living in the highest IMD crime areas reported on average 0.31 (95% CI -0.47, -0.15) fewer days of physical activity compared to those living in the lowest crime areas. Individuals living in the highest IMD crime areas achieved 5.56 fewer minutes of daily accelerometer-measured exercise than those in least crime areas.

**Conclusions:**

This study demonstrates consistent associations between perceived safety and objective crime with physical activity levels in adolescence.

